# Assessment of the Sun Nuclear ArcCHECK to detect errors in 6MV FFF VMAT delivery of brain SABR using ROC analysis

**DOI:** 10.1002/acm2.13276

**Published:** 2021-05-21

**Authors:** Sebastian Tattenberg, Derek Hyde, Marie‐Pierre Milette, Katia Parodi, Cynthia Araujo, Marco Carlone

**Affiliations:** ^1^ Department of Medical Physics Ludwig Maximilian University of Munich Garching Germany; ^2^ Irving K. Barber Faculty of Science University of British Columbia Okanagan Campus Kelowna BC Canada; ^3^ Centre for the Southern Interior Department of Medical Physics BC Cancer Agency Kelowna BC Canada

**Keywords:** ArcCHECK, PSQA, QA, ROC, SABR, VMAT

## Abstract

Institutions use a range of different detector systems for patient‐specific quality assurance (QA) measurements conducted to assure that the dose delivered by a patient’s radiotherapy treatment plan matches the calculated dose distribution. However, the ability of different detectors to detect errors from different sources is often unreported. This study contains a systematic evaluation of Sun Nuclear’s ArcCHECK in terms of the detectability of potential machine‐related treatment errors. The five investigated sources of error were multileaf collimator (MLC) leaf positions, gantry angle, collimator angle, jaw positions, and dose output. The study encompassed the clinical treatment plans of 29 brain cancer patients who received stereotactic ablative radiotherapy (SABR). Six error magnitudes were investigated per source of error. In addition, the Eclipse AAA beam model dosimetric leaf gap (DLG) parameter was varied with four error magnitudes. Error detectability was determined based on the area under the receiver operating characteristic (ROC) curve (AUC). Detectability of DLG errors was good or excellent (AUC >0.8) at an error magnitude of at least ±0.4 mm, while MLC leaf position and gantry angle errors reached good or excellent detectability at error magnitudes of at least 1.0 mm and 0.6°, respectively. Ideal thresholds, that is, gamma passing rates, to maximize sensitivity and specificity ranged from 79.1% to 98.7%. The detectability of collimator angle, jaw position, and dose output errors was poor for all investigated error magnitudes, with an AUC between 0.5 and 0.6. The ArcCHECK device’s ability to detect errors from treatment machine‐related sources was evaluated, and ideal gamma passing rate thresholds were determined for each source of error. The ArcCHECK was able to detect errors in DLG value, MLC leaf positions, and gantry angle. The ArcCHECK was unable to detect the studied errors in collimator angle, jaw positions, and dose output.

## 1 INTRODUCTION

Intensity‐modulated radiation therapy (IMRT) and volumetric modulated arc therapy (VMAT) treatments still frequently rely on patient‐specific QA measurements to ensure that the dose delivered to the detector using a patient’s treatment plan matches the expected dose distribution as calculated by the treatment planning system (TPS).^1^ These measurements can be performed with a variety of different detectors, including ionization chambers, diode arrays, radiochromic film, and portal imaging.^2^, ^3^, ^4^

The QA workflow is detector‐specific but, for systems like the ArcCHECK (Sun Nuclear Corporation, Melbourne, Florida, USA), it generally consists of re‐calculating the dose delivered by a patient’s treatment plan on the detector system and comparing it to a measurement to ensure accurate dose delivery.^5^, ^6^ Patient‐specific QA is essential for patient safety, especially in the case of a complex treatment delivery technique such as stereotactic ablative body radiotherapy (SABR), which delivers a high radiation dose in only a single or a few fractions and involves tight margins and often complex targets and beam geometries.^7^, ^8^ Systems like the ArcCHECK are useful for QA of conventional IMRT and VMAT plans as well as SABR treatments.^9^

A methodology commonly used for dose distribution comparisons is the gamma analysis method, which combines a distance‐to‐agreement (DTA) with a dose difference criterion to avoid inaccuracies in high‐gradient and low‐gradient regions, respectively.^10^, ^11^ Patient‐specific QA procedures use a previously set gamma passing rate threshold (e.g., 95%) to determine whether a sufficient percentage of points on the measured dose distribution agrees with the calculation.^1^ If this is not the case, the treatment plan fails the patient‐specific QA procedure, and the treatment cannot proceed with the plan in question before the reason for the failure has been determined and it has been established whether there is a need to revise the treatment plan. One of the shortcomings of reducing the gamma analysis results to a few metrics such as the passing rate is that such an approach does not allow the detector’s ability to identify errors originating from different sources to be taken into account.^11^

Receiver operating characteristic (ROC) analysis has previously been used to investigate a detector’s ability to detect treatment machine variations during plan delivery. In the case of the TrueBeam linear accelerator (Varian Medical Systems, Palo Alto, California, USA), such sources of error include the jaws which determine the size of the treatment field, the multileaf collimator (MLC) which conforms the radiation to the target, and the angle of the gantry.^12^ Studies using ROC analysis can fully evaluate the capabilities of a detector, including defining its rate of false positives and false negatives, that is, a detector wrongfully marking a plan as passing or failing because of its inability to accurately detect certain errors.^13^, ^14^ ROC curves are particularly useful for evaluating detector performance because they are independent of biases in the decision threshold which determines whether a plan passes or fails the QA procedure.^15^

Examples of ROC‐based error detectabiliy studies include research by Carlone et al.,^15^ McKenzie et al.,^16^ Bojechko & Ford,^17^ Nithiyanantham et al.,^18^ Liang et al.,^19^ Sjölin & Edmund,^20^ Maraghechi et al.,^21^ and Scarlet.^22^ However, combining the findings even of studies investigating the same detector can prove difficult because of limitations such as a small dataset, no differentiation between different treatment sites or delivery techniques, or some sources of error not having been studied. This study seeks to expand upon the aforementioned works by conducting a complete and systematic evaluation of the performance limits of a single detector — namely, Sun Nuclear’s ArcCHECK — in terms of its ability to detect expected machine‐related treatment errors in a set of brain VMAT SABR treatment plans using a 6 MV flattening filter free (6FFF) beam.

## 2 METHODS

### 2.1 Patient selection, treatment plan preparation, and delivery

Precise QA measurements are especially important for complex delivery techniques and relatively small targets and treatment fields.^7^ This study was based on the clinical treatment plans of patients treated with brain SABR, as these plans require particularly high precision and accuracy. The data set included the original treatment plans of 29 patients who received brain SABR at BC Cancer Kelowna. These clinical plans used 6 MV or the 6FFF mode of the Varian TrueBeam system. Since the 6FFF beam provides increased dose rates which can shorten the treatment time, which is beneficial when treatment fields are small and high doses are required, results for the 6FFF beam were of particular interest.^23^, ^24^ The clinical plans that used 6MV were therefore re‐planned using the 6FFF mode for this study.

An in‐house tool was used to anonymize all patient data, and the Varian Eclipse (V13) analytical anisotropic algorithm (AAA) was used to calculate the dose distributions delivered to the detector.^25^, ^26^ All treatment plans were delivered to Sun Nuclear’s ArcCHECK, which is a cylindrical polymethyl methacrylate (PMMA) phantom of a diameter of 21 cm with an array of 1386 SunPoint diodes on its surface.^27^ The same TrueBeam system and ArcCHECK detector were used for all measurements to prevent slight differences between different machines from influencing the results, and for a given source of error, all versions of a treatment plan were measured in the same session to avoid variations in the detector set‐up.

### 2.2 Gamma analysis

The calculated dose distributions were compared to the dose distributions measured on the surface of the ArcCHECK using the gamma analysis approach as implemented in Version 6.2.3 of Sun Nuclear’s SNC Patient software.^10^, ^28^ All studies were repeated for three different sets of criteria: 2%/2 mm, 2%/1 mm, and 4%/1 mm. The 2%/2 mm criteria were chosen in accordance with the planning target volume (PTV) margin of 2 mm, while the other two criteria were added to study the effects of variations in the dose difference or the DTA criterion. A threshold of 10% was used below which dose values were disregarded.

### 2.3 Determination of the consensus optimal dosimetric leaf gap value

The Eclipse AAA model uses a dosimetric leaf gap (DLG) parameter to model the leakage through the curved edges of the MLC leaves.^29^, ^30^ However, the clinically‐used DLG value is determined for a broad set of patients and treatment sites, and differences of up to 0.8 mm between the clinical and the plan‐specific optimal DLG value have been reported.^22^ Due to the high‐precision requirements for the clinical 6FFF beam, the optimal DLG value for brain SABR treatment planning had to be determined.

Nine representative brain SABR treatment plans were delivered to a cylindrical ionization chamber (Scanditronix Wellhöfer Dosimetrie, Schwarzenbruck, Germany), EBT3 Gafchromic film (Ashland Inc., Covington, Kentucky, USA), and the ArcCHECK to determine as accurate an optimal DLG value as possible. For the ionization chamber, the difference between the measured dose and the calculated dose was plotted as a function of the DLG value and the position of the minimum difference was defined as the optimal DLG value. For film and the ArcCHECK, the optimal DLG value was defined as the value that maximized the gamma passing rates.^31^, ^32^ All three methods yielded the same consensus optimal DLG value of 1.47 mm. This value was very close to the clinical value used at our institution, which is 1.40 mm.

### 2.4 Implementation of machine‐related treatment errors

The investigated sources of error were the DLG value, the MLC leaf positions, the gantry angle, the collimator angle, the jaw positions, and the dose output. The latter five were investigated because they were specifically mentioned as potential sources of error in the specifications of Varian’s TrueBeam system, while the former was included because the plan‐specific optimal DLG value is known to commonly differ from the value used in the clinical context, which is determined for and applied to a vast range of treatment sites.^12^, ^33^ Simultaneous errors from different sources lay outside the scope of this study because of the sheer number of possible permutations and because such studies would not help quantify the ArcCHECK’s limits with respect to the detectability of errors from a given source.

The sources of error which necessitated measurements of modified treatment plans were MLC leaf positions, gantry angle, collimator angle, and jaw positions. The original treatment plans were exported from the treatment planning system in the Digital Imaging and Communications in Medicine (DICOM) format, and a MATLAB (MathWorks, Natick, Massachusetts, USA) script was used to introduce errors from different sources and of different magnitudes into different copies of the original treatment plans. Six modified treatment plans with different error magnitudes were created for each of these four sources of error, so that for each of the 29 patient plans, a total of 25 different treatment plans existed: the original unmodified plan, and six additional versions into which errors of different magnitudes had been introduced into the MLC leaf positions, the gantry angle, the collimator angle, or the jaw positions. This resulted in a total of 29 × 4 × 7 = 812 measurements, with the unmodified treatment plans being measured for every study to prevent differences in the detector set‐up from influencing the results. Four DLG error magnitudes were also investigated but only required measurements of the unmodified treatment plans, which were already conducted for all other types of error. The detectability of six dose output error magnitudes was approximated in a way which also only relied on measurements of the unmodified treatment plans. This methodology yielded 5 × 6 + 1 × 4 = 34 ROC curves for each of the three studied gamma analysis criteria, for a total of 3 × 34 = 102 ROC curves.

The magnitude of the errors introduced into the plans were based on the specifications of Varian’s TrueBeam system, with additional higher error magnitudes being investigated to account for other realistic scenarios and to test the capabilities of the ArcCHECK. In the case of MLC leaf position errors, for example, Varian’s HD120 MLC specifications state a leaf end positional accuracy of ±1.0 mm.^12^ Errors of up to ±1.5 mm were investigated nonetheless because errors of such magnitudes have been observed in Varian’s Clinac iX system.^34^, ^35^

#### 2.4.1 MLC leaf position errors

In the underlying DICOM file, each SABR treatment plan is divided into multiple arcs, each of which is in turn discretized into dozens of control points. The treatment delivery system delivers the treatment plan by setting the collimator angle and jaw positions to the values specified for each arc and delivering the specified amount of radiation at every control point after having set the gantry angle and MLC leaf positions to the control point’s values.

To determine the detectability of MLC leaf position errors, the leaf positions at every control point were modified with random errors of up to ±0.25 mm, ±0.50 mm, ±0.75 mm, ±1.00 mm, ±1.25 mm, and ±1.50 mm, with the six different maximum error magnitudes representing six different sets of 29 modified treatment plans each. The mean values and standard deviations for the six resulting distributions were 0.15 mm ± 0.01 mm, 0.27 mm ± 0.03 mm, 0.38 mm ± 0.03 mm, 0.55 mm ± 0.06 mm, 0.64 mm ± 0.05 mm, and 0.87 mm ± 0.07 mm. Random errors were chosen over systematic ones because systematic errors are typically corrected during routine machine QA. All modified treatment plan files were loaded into the TrueBeam system and delivered to the ArcCHECK device.

#### 2.4.2 Gantry angle errors

To investigate the detectability of gantry angle errors, random errors of up to ±0.15°, ±0.30°, ±0.45°, ±0.60°, ±0.75°, and ±0.90° were introduced into the gantry angle position at every control point for the six different sets of 29 modified treatment plans. The mean values and standard deviations for the six resulting distributions were 0.075° ± 0.002°, 0.150° ± 0.005°, 0.223° ± 0.006°, 0.301° ± 0.009°, 0.374° ± 0.014°, and 0.454° ± 0.014°.

#### 2.4.3 Collimator angle errors

To determine the detectability of collimator angle errors, random errors of between 0.00° to ±0.25°, ±0.25° to ±0.50°, ±0.50° to ±0.75°, ±0.75° to ±1.00°, ±1.00° to ±1.25°, and ±1.25° to ±1.50° were introduced into the six sets of modified treatment plans. Collimator angle errors were forced to be within a range rather than being completely random because the low number of modifiable collimator angles could otherwise have led to solely small errors being introduced into plans which were supposed to exhibit large collimator angle errors. This was because each plan had dozens or hundreds of control points with modifiable MLC leaf positions and gantry angles, while the collimator angle and jaw positions were specified for an entire arc, of which each plan only had a few. For the six different collimator angle error ranges, the mean values and standard deviations for the resulting distributions of errors introduced into the six different sets of treatment plans were 0.12° ± 0.07°, 0.37° ± 0.07°, 0.62° ± 0.07°, 0.87° ± 0.08°, 1.13° ± 0.08°, and 1.37° ± 0.08°.

#### 2.4.4 Jaw position errors

Errors in the upper and lower jaw were studied jointly, with six different “error levels” being defined for this purpose. Per error level, each end of the range of possible errors increased by 0.5 mm for the upper jaw and by 0.25 mm for the lower jaw. Error level 1 therefore corresponded to errors ranging from 0.0 mm to 0.5 mm in the upper jaw and errors between 0.00 mm and 0.25 mm in the lower jaw position. For error level 2, errors in the upper jaw ranged from 0.5 mm to 1.0 mm, while errors in the position of the lower jaw ranged from 0.25 mm to 0.50 mm. Higher error levels were defined accordingly. For a given error level, the magnitude of upper and lower jaw position errors differed in accordance with the TrueBeam specifications, which state a worse positional accuracy for the upper than the lower jaw.^12^ As in the case of the collimator angle, jaw position errors were forced to be in a range rather than being completely random to assure that errors of the studied magnitudes were actually introduced into the treatment plans.

For the upper jaw, the mean values and standard deviations of the six different error value distributions were 0.23 mm ± 0.15 mm, 0.74 mm ± 0.15 mm, 1.21 mm ± 0.14 mm, 1.75 mm ± 0.16 mm, 2.26 mm ± 0.15 mm, and 2.75 mm ± 0.15 mm. For the position of the lower jaw, the corresponding values were 0.12 mm ± 0.07 mm, 0.39 mm ± 0.07 mm, 0.61 mm ± 0.07 mm, 0.88 mm ± 0.07 mm, 1.13 mm ± 0.07 mm, and 1.38 mm ± 0.07 mm.

#### 2.4.5 DLG errors

As the dosimetric leaf gap is solely a TPS parameter, determining the detectability of DLG value errors did not require additional measurements. Instead, four additional dose calculations were run for every unmodified treatment plan. These calculations used DLG values deviating from the previously determined consensus optimal DLG value by −0.4 mm, −0.2 mm, +0.2 mm, and +0.4 mm. These error magnitudes were deemed to be realistic because they were in line with DLG value errors which have been reported previously.^22^

#### 2.4.6 Dose output errors

Dose output errors did not require further measurements either because their detectability was approximated using changes in the “dose per count” value of the *.txt file created by the measurement of an unmodified treatment plan. The six different maximum possible error magnitudes studied were 0.25%, 0.50%, 0.75%, 1.00%, 1.25%, and 1.50%. The mean values and standard deviations for the resulting distributions were 0.16% ± 0.08%, 0.36% ± 0.07%, 0.62% ± 0.08%, 0.85% ± 0.08%, 1.13% ± 0.06%, and 1.37% ± 0.07%.

### 2.5 Receiver operating characteristic curves

Receiver operating characteristic curves are analytical tools for the evaluation of a diagnostic test which outputs binary results.^36^, ^37^, ^38^ The acceptance threshold (in this work, the gamma passing rate) which decides whether a result is considered a positive or a negative is tuned, and the ROC curve is created by plotting the false positive fraction (which is equal to 1‐specificity) on the x‐axis against the sensitivity on the y‐axis for every acceptance threshold. To quantify the ArcCHECK’s ability to detect errors from a given source and of a given magnitude, the area under the ROC curve (AUC) was used, with a value of 0.5 being equal to random guessing and a value of 1.0 denoting perfect detectability.^37^

ROC curves were created in Version 7 of Prism (GraphPad Software, San Diego, California, USA), with every ROC curve being based on two sets of gamma passing rates: one for which the measurements of the unmodified treatment plans had been compared to calculations using the consensus optimal DLG value (the gold standard), and one for which either the measured treatment plans, the DLG values, or the output files had previously had errors introduced into them. The different points on the ROC curve were yielded through tuning of the threshold value, and the optimal threshold value, that is, the value at which the distance between the ROC curve and the point of perfect sensitivity and specificity (0,1) was minimal, was determined for all sources of error which exhibited sufficient detectability.^15^ The ways in which the gold standard and the evaluated data set were defined for the different sources of error are shown in Table 1.

**Table 1:** Definitions of reference (gold standard) and evaluated gamma indices.

Source of error	Evaluated gamma indices	Reference gamma indices
Dosimetric leaf gap	Measurements of unmodified treatment plans Calculations using modified DLG values	Measurements of unmodified treatment plans Calculations using consensus optimal DLG value
MLC leaf positions Gantry angle Collimator angle Jaw positions	Measurements of modified treatment plans Calculations using consensus optimal DLG value	
Dose Output	Modified measurements of unmodified treatment plans Calculations using consensus optimal DLG value	

The ways in which the reference (gold standard) and evaluated sets of gamma indices from which the ROC curves were created were defined for the different sources of error.

## 3 RESULTS

### 3.1 DLG value errors

**Fig. 1:**
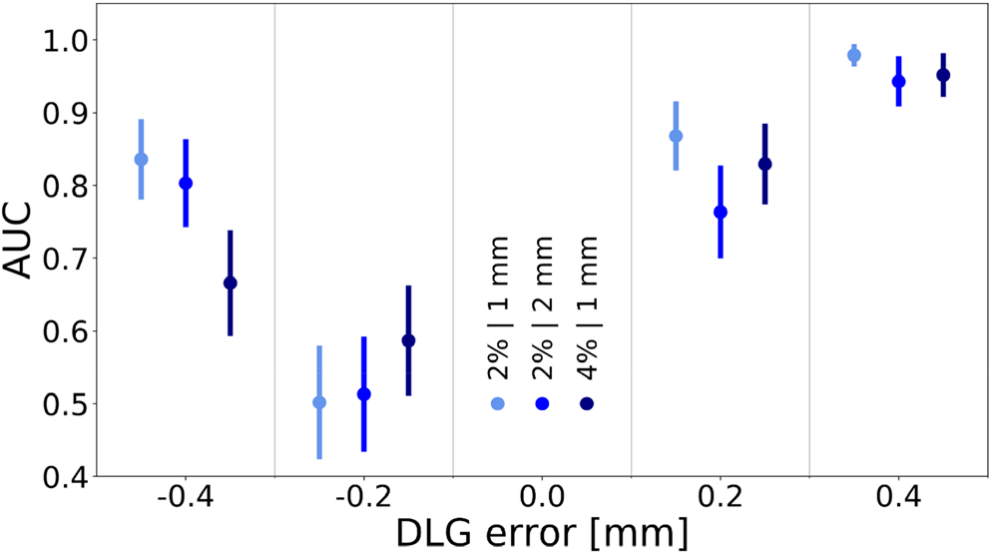
The area under the ROC curve as a function of the error introduced into the dosimetric leaf gap value. The data points on the left, in the middle, and on the right of each column denote the 2%/1 mm, 2%/2 mm, and 4%/1 mm criterion, respectively. Error bars indicate the standard error.

The results regarding the detectability of DLG value errors are depicted in Fig. 1. The AUC values indicated that detectability was very good when the DLG value was 0.4 mm higher than the consensus optimal DLG value. Detectability of DLG errors of −0.4 mm and +0.2 mm was decent, and the detectability of a DLG value 0.2 mm lower than the consensus optimal DLG value was poor.

**Fig. 2:**
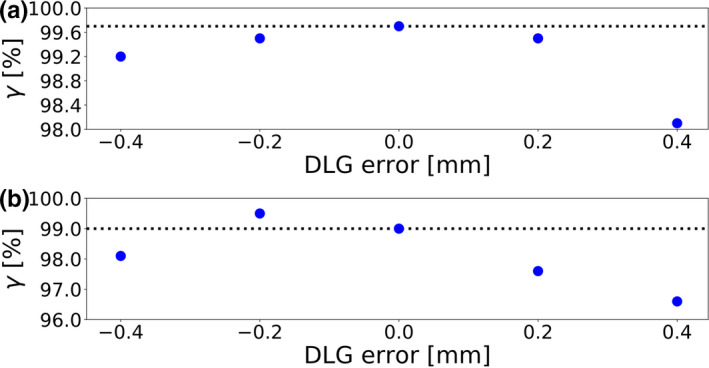
The gamma passing rate (2%/2 mm) as a function of the error introduced into the dosimetric leaf gap value for a case for which the plan‐specific optimal DLG value was equal to the consensus optimal DLG value determined for a representative set of nine treatment plans (a) and a case for which the plan‐specific optimal DLG value likely differed from the consensus value by −0.2 mm (b). The dotted lines indicate the gamma passing rate of the gold standard.

To emphasize how differences in the plan‐specific optimal DLG values influenced the detectability of DLG errors, Fig. 2 depicts the gamma passing rate as a function of the DLG error for two cases — one in which the plan‐specific optimal DLG value was equal to the consensus value, and one in which this was likely not the case.

On a single‐plan basis and at low error magnitudes, error detectability can roughly be approximated by the difference between the gamma passing rate at the gold standard and at the error magnitude in question. In cases like the one shown in Fig. 2(a), in which the consensus value was equal to the plan‐specific optimal DLG value, the gamma passing rates at error magnitudes of −0.2 mm and +0.2 mm were approximately equal. Such cases generally contributed to a similar detectability of negative and positive DLG errors of the same magnitude.

In the case shown in Fig. 2(b), on the other hand, the ArcCHECK measurement indicated that the plan‐specific optimal DLG value may have differed from the consensus value by about −0.2 mm. In this case, the gamma passing rate of the gold standard was much closer to the gamma passing rate at an error magnitude of −0.2 mm than at an error magnitude of +0.2 mm, contributing to a reduced detectability of the −0.2 mm error. For the 4%/1 mm criterion, the plan‐specific optimal DLG values were likely lower than in the case of the other criteria, leading to a more pronounced asymmetry in the data. The severity of the effect at an error magnitude of −0.2 mm, at which error detectability was poor for all criteria, indicates plan‐specific optimal DLG values that frequently differed from the consensus value by approximately −0.2 mm.

### 3.2 MLC leaf position errors

**Fig. 3:**
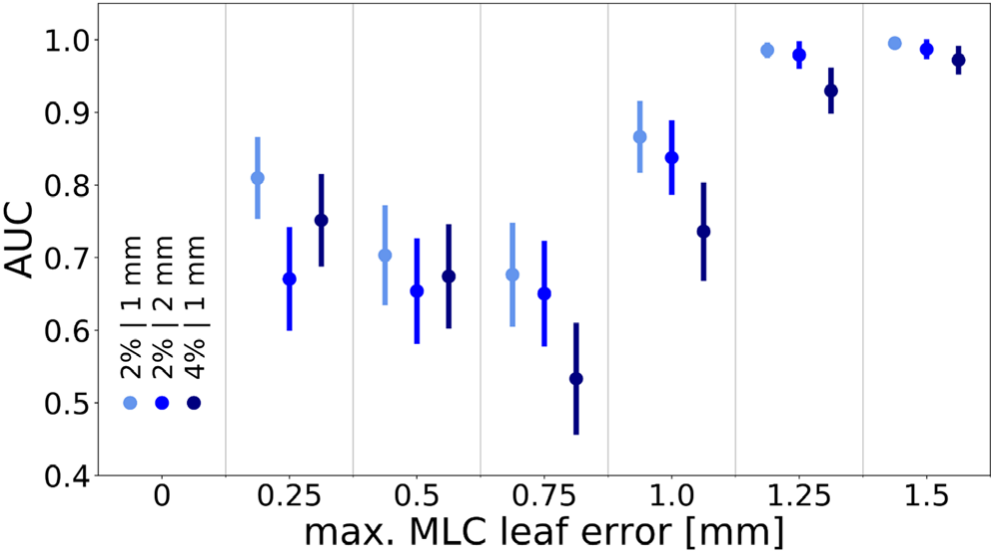
The area under the ROC curve as a function of the error introduced into the multileaf collimator leaf positions. The data points on the left, in the middle, and on the right of each column denote the 2%/1 mm, 2%/2 mm, and 4%/1 mm criterion, respectively. Error bars indicate the standard error.

The results of the MLC leaf position error detectability study are shown in Fig. 3. The data exhibited variations in the detectability of MLC leaf position errors of 0.75 mm or lower, especially for the 2%/1 mm and 4%/1 mm criterion. This was again caused by the planning system modeling of the MLC leaf ends, which uses a single DLG value.

**Fig. 4:**
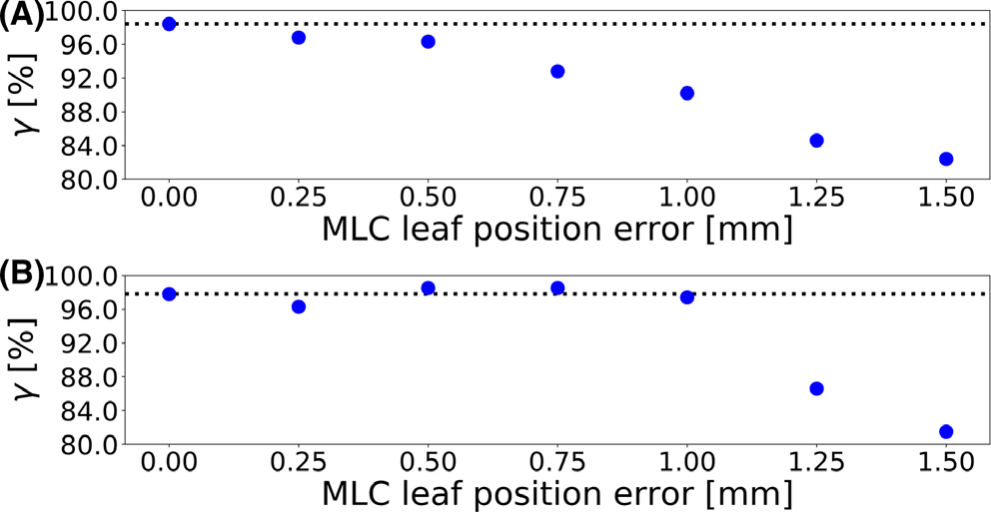
The gamma passing rate (2%/2 mm) as a function of the multileaf collimator leaf position error for two cases: (a) a plan for which the plan‐specific optimal DLG value was equal to the consensus optimal DLG value determined for a representative set of nine treatment plans and (b) a plan for which the plan‐specific optimal DLG value likely differed from the consensus value. The dotted lines indicate the gamma passing rate of the gold standard.

To elaborate on this point, Fig. 4 depicts the gamma passing rate as a function of the maximum MLC leaf position error for a case in which the plan‐specific DLG value was equal to the consensus value and an example in which this was likely not the case. The leakage through the curved MLC leaf edges is simulated by the position of every MLC leaf being retracted by half of the DLG value.^33^ In cases like the one depicted in Fig. 4(a), in which the plan‐specific optimal DLG value was equal to the consensus value, the highest gamma passing rate was reached when the plan was unmodified. In such cases, the gamma passing rate decreased with increasing MLC leaf position errors, contributing to a higher AUC and better error detectability at higher MLC leaf position error magnitudes.

In cases like the one depicted in Fig. 4(b), in which the ArcCHECK measurement suggested that the plan‐specific optimal DLG value differed from the consensus value, the gamma passing rate was not necessarily highest when the plan was unmodified. Instead, plans with errors in the MLC leaf positions were able to match the dose distribution calculated using a DLG value that was likely not the plan‐specific optimal value more closely. In such cases, the gamma passing rate peaked when MLC leaf position errors were introduced into the plan, contributing to an uneven relationship between MLC leaf position error magnitude and detectability. In the case of the 2%/2 mm criterion, for which the consensus value tended to be correct for most plans, the detectability of MLC leaf position errors was consistent and relatively poor for all error magnitudes lower than 1.00 mm. For all criteria, the detectability of MLC leaf position errors improved at higher magnitudes. The detectability of MLC leaf position errors of 1.00 mm was generally decent, and MLC leaf position errors of up to 1.25 mm or more were detected excellently.

### 3.3 Gantry angle errors

**Fig. 5:**
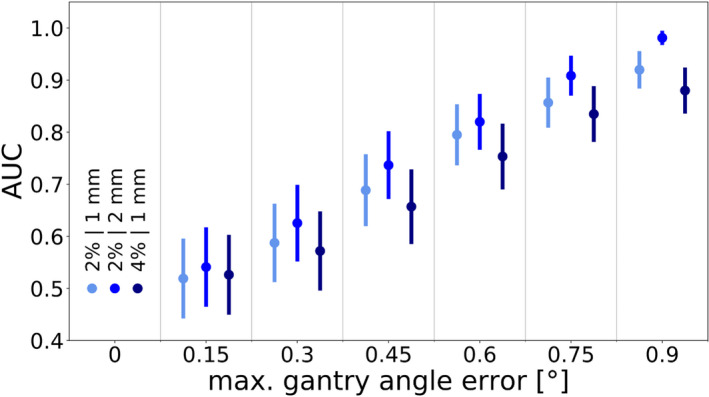
The area under the ROC curve as a function of the error introduced into the gantry angle. The data points on the left, in the middle, and on the right of each column denote the 2%/1 mm, 2%/2 mm, and 4%/ 1 mm criterion, respectively. Error bars indicate the standard error.

The results regarding the detectability of gantry angle errors are shown in Fig. 5. In this case, the expected trend of the area under the ROC curve increasing with higher gantry angle errors was observed for all criteria, with a generally good detectability in cases with a maximum possible gantry angle error of at least 0.6°.

### 3.4 Collimator angle, jaw position, and output errors

For errors in the collimator angle, jaw positions, and output, detectability was poor for all criteria and all investigated error magnitudes, with an AUC around or below 0.6 in all cases.

### 3.5 Optimal threshold values

For the sources of error for which the ArcCHECK exhibited an ability to detect errors of a given magnitude — namely, the DLG value, the MLC leaf positions, and the gantry angle — the optimal threshold values (i.e., gamma passing rates) for all investigated error magnitudes and criteria are shown in Table 2. For the 2%/2 mm, the 2%/1 mm, and the 4%/1 mm criterion, the ideal threshold for errors in the DLG value ranged from 96.9% to 98.2%, from 82.7% to 88.4%, and from 91.1% to 95.1%, respectively. For errors in the MLC leaf positions, the corresponding ranges were 96.9% to 98.7%, 79.1% to 85.5%, and 91.1% to 95.5%, while for errors in the gantry angle, ideal thresholds ranged from 97.3% to 98.2%, from 79.5% to 83.4%, and from 88.7% to 94.3%. At low error magnitudes, some of the determined optimal threshold values exhibited small inconsistencies in the form of a slight increase in the threshold value for an increase in the error magnitude which were in line with the inconsistencies in error detectability described above.

**Table 2:** ideal threshold values for different sources of error and error magnitudes.

Source of error	Error magnitude	Ideal threshold for criterion [%]		
		2%/2 mm	2%/1 mm	4%/1 mm
DLG	−0.4 mm	97.6	82.7	95.1
	−0.2 mm	97.2	88.4	95.1
	0.2 mm	98.2	85.8	93.2
	0.4 mm	96.9	82.7	91.1
MLC	0.25 mm	97.6	85.8	94.1
	0.50 mm	98.2	85.8	95.1
	0.75 mm	98.7	85.8	95.5
	1.00 mm	97.7	83.6	94.1
	1.25 mm	96.9	79.1	91.1
	1.50 mm	96.9	79.1	91.1
Gantry angle	0.15°	98.2	81.8	94.3
	0.30°	98.1	82.5	92.2
	0.45°	97.3	83.4	92.1
	0.60°	97.7	80.5	91.2
	0.75°	97.3	80.5	91.2
	0.90°	97.3	79.5	88.7

The determined optimal threshold values for errors in the DLG value, the MLC leaf positions, and the gantry angle as a function of error magnitude.

## 4 DISCUSSION

The ArcCHECK’s ability (or lack thereof) to detect errors stemming from different sources results from its technological characteristics and limitations. Its 1386 diodes have an active detection area of 0.64 mm^2^ each, with a detector spacing of 1.0 cm.^27^ Detector systems with more favorable characteristic such as smaller spacing between detector elements are expected to provide superior error detectability, with the degree of benefit depending on the source of error in question.

### 4.1 DLG value errors

The asymmetry in the results regarding DLG error detectability was due to the Eclipse AAA model using a single DLG value to model the MLC leaf ends. This is a deficiency in the MLC modeling of the AAA algorithm and can be corrected by determining a plan‐specific DLG value for each plan, but doing so would be infeasible in the clinic. Due to some plan‐specific optimal DLG values likely being lower than the determined consensus optimal DLG value, the detectability of DLG errors of −0.2 mm was poor. This aspect also caused DLG errors of −0.4 mm and +0.2 mm to exhibit a similar level of detectability, which was decent to good. Only DLG errors of +0.4 mm were detected excellently. This is to be considered in light of the magnitude of realistic DLG errors, and differences of up to 0.8 mm between the clinically used and the plan‐specific optimal DLG value have been reported.^22^ The ArcCHECK device is therefore able to detect medium to high DLG errors which may realistically be encountered in the clinical context. Detecting such errors in a number of cases may indicate that the clinical DLG value used is inaccurate for the cases to which it is applied and needs to be corrected.

### 4.2 MLC leaf position errors

The detectability of MLC leaf position errors of 0.75 mm and smaller was highly dependent on how close the plan‐specific optimal DLG value was to the optimal DLG value used in the calculation. Since the DLG parameter is used to model the MLC, it is not surprising that the detection of MLC leaf positioning errors is affected by how appropriate the DLG value is for a specific plan. Despite this effect, none of the data indicated a good detectability of MLC leaf position errors of up to 0.75 mm or smaller. Only beginning at maximum MLC leaf position errors of 1.00 mm did detectability improve. When MLC leaf position errors were allowed to reach up to 1.25 mm and 1.50 mm, detectability was excellent. This is in line with a previous study, which was based on the treatment plans of seven prostate patients who received VMAT and which found that the ArcCHECK was able to detect MLC leaf position errors of 1 mm under similar circumstances when a 2%/2 mm criterion was used.^21^ The same study found the ArcCHECK to be inferior to the electronic portal imaging device (EPID) also tested in terms of MLC leaf position error detectability. The superiority of EPID systems with regards to the detectability of MLC leaf position errors was confirmed by a study which introduced systematic MLC leaf position errors into IMRT and VMAT treatment plans and which used an EPID with a spatial resolution of 0.392 mm.^39^ This outcome is in line with the expectation of systems with a higher spatial resolution having an advantage in terms of error detectability.

With MLC leaf position errors of up to 1.00 mm being expected in Varian Clinac iX and TrueBeam systems and errors of up to 1.50 mm having been reported, the ArcCHECK should be able to detect MLC leaf position errors on the higher end of these reasonably expected magnitudes.^12^, ^35^, ^36^

### 4.3 Gantry angle errors

The detectability of gantry angle errors followed the expected trend of improving with increasing gantry angle error magnitudes, and the data for all three investigated criteria showed good agreement. For all criteria, the detectability of gantry angle errors of up to 0.45° was decent at best, with AUCs of 0.7 or lower, and the detectability of gantry angle errors of up to 0.60° was decent to good. At gantry angle errors of up to 0.75° and up to 0.90°, error detectability was good or excellent, with AUCs of around 0.8 to 0.9 and only small differences on the scale of the standard error being observed between the different criteria.

The ArcCHECK’s advantage over systems like Delta^4^ (ScandiDos, Uppsala, Sweden) and an EPID in terms of gantry angle error detectability has previously been reported in a study based on a set of VMAT plans for head and neck patients.^19^ In the aforementioned study, the AUC of 0.78 for a gantry angle error magnitude of 1° was still associated with good error detectability but was lower than the error detectability determined as part of this work. The differences between the results of the two studies could be explained by factors such as the different treatment sites, the different types of treatment, and the different ways in which the errors were implemented, amongst others. For example, the error magnitudes in the VMAT study were a function of the gantry angle while the gantry angle errors investigated for this study were random.

Whether higher magnitudes of gantry angle errors are realistic is questionable. Varian’s TrueBeam system, for instance, states a rotational gantry accuracy ≤0.3°, which the ArcCHECK would not be able to detect.^12^ However, if a different delivery system was used or larger gantry angle errors were anticipated for other reasons, the ArcCHECK may be able to detect gantry angle errors relatively well.

### 4.4 Collimator angle errors

For all criteria, the ArcCHECK’s ability to detect collimator angle errors of any of the studied magnitudes was poor. This is true despite the introduced collimator angle errors having been forced to be within a range to assure that errors of the studied magnitudes were actually introduced into the treatment plans. Because of the magnitude of the standard error, the small differences between the AUC values at different collimator angle error magnitudes were negligible.

The ArcCHECK’s perceived inability to detect collimator angle errors was hinted at by a previous study, which showed that a collimator angle error of 1° only changed the gamma passing rate of a brain and a head and neck VMAT treatment plan by 0.3% and 1.6%, respectively, when a 2%/2 mm criterion was used.^40^ Since systems like Varian’s TrueBeam claim a rotational accuracy of ≤0.5° for the collimator and collimator angle error magnitudes of up to 1.5° were investigated, the ArcCHECK is unable to detect collimator angle errors of the magnitudes one may generally expect to encounter.^12^

### 4.5 Jaw position errors

For all considered criteria, the detectability of jaw position errors of all investigated magnitudes was also poor. Once again, the small differences between data points at the different error levels were negligible compared to the size of the standard error. The ArcCHECK’s poor detectability of jaw position errors has also been indicated by a previous study, which introduced an error of 3 mm into the Y1 jaw position of a brain and a head and neck VMAT treatment plan and only reported gamma passing rate decreases of 0.1% and 0.0%, respectively, when a 2%/2 mm criterion was used.^40^

The specifications of Varian’s TrueBeam system suggest an upper jaw positional accuracy of ±2 mm and a lower jaw positional accuracy of ±1 mm for static fields.^12^ The highest investigated error magnitudes of ±3 mm and ±1.5 mm, respectively, exceeded these values, and errors in the upper and lower jaw were investigated together, meaning that the highest jaw position error level corresponded to the worst‐case scenario regarding the accuracy of both the upper and lower jaw position. Despite these considerations, none of the criteria suggested even decent detectability at any error level. The ArcCHECK’s ability to detect realistic jaw position errors in either jaw in the studied brain SABR treatment plans can therefore be regarded as being poor.

### 4.6 Dose output errors

Independently of the criterion used, the ArcCHECK was not able to detect dose output errors of any of the investigated magnitudes, with an AUC of approximately 0.6 or lower at all error magnitudes, even though the highest such errors were larger than the uncertainty of systems such as Varian’s TrueBeam.^12^ Dose output errors were simulated through modifications of the measurement files rather than being investigated through measurements of modified treatment plans. Prior to choosing this approach, sample measurements confirmed that the scaling of the “dose per count” value was equivalent to the measurement of a modified treatment plan, but comparisons to confirm this were not run for all 29 cases included in this study. However, the highest dose output error magnitude studied was 1.5%, whereas the dose difference criteria used were 2%, 2%, and 4%. As the dose output error magnitude was always within the dose difference criterion, the detectability of the studied dose output errors was not necessarily expected to be good, even though such errors affect the entire dose distribution. This is also in line with the results of a previous study, which reported the ArcCHECK’s inability to detect even output errors of 5% in a set of VMAT head and neck treatment plans.^19^ The same study also showed that Delta^4^ and an EPID were equally unable to detect the same output errors. It was therefore concluded that the ArcCHECK does not detect output errors of up to 1.5% with any reliability when using the studied criteria.

### 4.7 Clinical implications

The ArcCHECK’s capabilities with respect to the detectability of errors from different sources as determined by this study constitute its limits rather than what would necessarily be expected to be observed in clinical practice at every institution. This is because the gold standard dose calculations made use of the optimal DLG value determined specifically for brain SABR treatment plans rather than a compromised value which is often used clinically. This approach was chosen because it allowed the ArcCHECK’s limits with regards to error detectability to be established, rather than yielding results which are strictly dependent on the accuracy of the DLG value used at a given institution. The optimal DLG value determined as part of this study differed from the clinical value of 1.40 mm by only 0.07 mm, compared to deviations of up to 0.80 mm reported elsewhere.^22^ The ArcCHECK’s ability to detect errors in the clinical context of the institution at which the study was conducted would therefore be expected to be similar to the limits established in this study. If the deviation between the clinical and the optimal DLG value was larger, however, the ArcCHECK would be expected to exhibit poorer error detectability. Error detectability was generally found to be consistent for all three investigated gamma analysis criteria. The criteria used at a given institution are therefore generally not expected to affect error detectability. The ideal threshold values (i.e., the gamma passing rates maximizing sensitivity and specificity when analyzing 6FFF brain SABR plans) determined as part of this study may be used to improve the analysis of detector measurements.

## 5 CONCLUSION

Of the investigated machine‐related sources of error, the ArcCHECK detected errors in the MLC leaf positions most reliably. Its ability to detect MLC leaf positions errors of at least 1.0 mm, which lie within the tolerance limits of systems such as the Varian TrueBeam, was generally good or excellent (AUC >0.85). Errors in the gantry angle were only detected well (AUC >0.80) if the error magnitude was at least 0.6°, which would be twice as high as the maximum errors generally expected in TrueBeam systems. DLG error detection was generally good or excellent for error magnitudes of at least ±0.4 mm (AUC >0.80). The detectability of errors in the collimator angle, the upper and lower jaw position, and the dose output, on the other hand, was poor. Such errors would therefore have to lie far outside the TrueBeam’s tolerance limits to potentially be detected by the ArcCHECK. Using a generalized DLG parameter in the underlying dose calculations is expected to negatively affect error detectability. Ideal threshold values (i.e., gamma passing rates) which may be used to optimize the analysis of detector measurements were also determined.

## Conflicts of Interest

No conflicts of interest.

## Author Contribution

Because of his previous experience in the area, M.C. conceived the idea behind the work. All authors contributed to its design and the interpretation of the data. Measurements were conducted and the resulting data were analyzed by S.T. after extensive instruction by C.A. All authors contributed to the drafting of the work and subsequent revisions and approved the final version prior to submission.

## Data Availability

The data that support the findings of this study are available from the corresponding author upon reasonable request.
